# Protective Potential of *Limosilactobacillus fermentum* Strains and Their Mixture on Inflammatory Bowel Disease via Regulating Gut Microbiota in Mice

**DOI:** 10.4014/jmb.2410.10009

**Published:** 2024-12-10

**Authors:** Jae Yeon Joung, Kayoung Choi, Ju-Hoon Lee, Nam Su Oh

**Affiliations:** 1 Department of Food and Animal Biotechnology, Seoul National University, Seoul 08826, Republic of Korea; 2 Department of Agricultural Biotechnology, Seoul National University, Seoul 08826, Republic of Korea; 3 Center for Food and Bioconvergence, Seoul National University, Seoul 08826, Republic of Korea; 4 Research Institute of Agriculture and Life Science, Seoul National University, Seoul 08826, Republic of Korea; 5 Department of Food and Biotechnology, Korea University, Sejong 30019, Republic of Korea

**Keywords:** Inflammatory bowel disease, anti-inflammation, gut microbiota, probiotics, *Limosilactobacillus fermentum*

## Abstract

The aim of this study is to investigate the protective potential of *Limosilactobacillus fermentum* IM57, IR51, and IR62 strains, isolated from infant feces, and their mixture against inflammatory bowel disease (IBD). The strains exhibited robust antioxidant activities and anti-inflammatory properties in RAW 264.7 cells. Subsequently, the potential protective effects of each of these three strains, along with their mixture, were evaluated in a murine colitis model induced by dextran sodium sulfate (DSS). Noteworthy improvements in physiological parameters such as body weight, disease activity index, and colon length were observed in mice treated with the mixture followed by IR62. Additionally, administration of each strain and the mixture mitigated DSS-induced changes in gut microbiota composition with increased abundance of *Lactobacillus*, *Bifidobacterium*, *Ruminococcus*, and *Muribaculum*, compared to DSS-treated mice. Interestingly, the abundance of *Muribaculum* increased approximately 2.4-fold after administration of the mixture compared to before administration. Additionally, the concentration of short-chain fatty acids (SCFAs) was significantly reduced in DSS-treated group compared to the control group, while the mixture treatment group had the highest concentration of SCFAs. Furthermore, due to these changes in microbiota and the leading metabolites induced by treatment of the mixture, DSS-induced dysregulation of inflammationand barrier function-related mRNA expressions was significantly inhibited in the group fed with the mixture. Consequently, this study indicates that the multi-strain mixture of *L. fermentum* strains may play a crucial role in modulating gut microbiota, thereby alleviating IBD through the synergistic effect of the individual effects of the three strains.

## Introduction

Inflammatory bowel disease (IBD), encompassing ulcerative colitis (UC) and Crohn’s disease, is a chronic inflammatory disorder of the gastrointestinal tract. The global prevalence of IBD has risen in recent years, affecting approximately 4.9 million individuals worldwide, with 41,000 associated deaths as reported by the Global Burden of Disease in 2019 [1]. The etiology of IBD is not fully understood; however, it is believed to involve complex interactions between genetic factors, intestinal barrier function, immune responses, and the gut microbiome [2]. Notably, several hypotheses propose that IBD arises from immune system dysregulation due to chronic microbiota imbalance in the gastrointestinal tract [3]. While it was initially thought to be driven predominantly by adaptive immunity, recent studies have highlighted the significant role of innate immunity in disrupting the composition of gut microbiota [4]. The unpredictable nature of IBD complicates its management. Conventional treatment strategies aim to control symptoms through the use of medications such as aminosalicylates (*e.g.*, mesalamine), immunomodulators (*e.g.*, azathioprine), and biologics (*e.g.*, infliximab). Mesalamine, also known as mesalazine or 5-aminosalicylic acid (5-ASA), is a commonly prescribed anti-inflammatory drug for alleviating IBD symptoms. However, more than half of IBD patients experience a secondary loss of response and reduced efficacy to 5-ASA over time [5]. To address the limitations of current IBD treatments, the development of alternative or supplementary therapies has been proposed. Combining traditional IBD medications with supplements may enhance therapeutic outcomes. Among the supplements evaluated for IBD treatment, probiotics have been frequently investigated for their potential benefits [6].

Probiotics, including strains such as *Lactobacillus*, *Bifidobacterium*, *Streptococcus*, and *Enterococcus*, are known to confer health benefits to the host, as reported by the FAO/WHO (2006). These benefits include the alleviation of diarrhea and constipation, reduction of lactose intolerance, immune regulation, anti-tumorigenic effects, and cholesterol reduction [7]. Some probiotic strains have been clinically evaluated for their efficacy in treating IBD, due to their ability to modulate immune function [7]. The rationale for using probiotics in IBD management is based on their well-documented biological mechanisms, which include: (a) antioxidant and anti-inflammatory properties, (b) restoration of the gut barrier and mucosal layers in epithelial cells, and (c) rebalancing of the gut microbiota to promote gut homeostasis and short-chain fatty acid (SCFA) production (Ref). To evaluate these mechanisms in IBD alleviation, various in vivo animal and human studies have been conducted, focusing on the following aspects:

(a) Antioxidant and Anti-inflammatory Properties: Chen *et al*. [8] reported that administering *L. fermentum* ZS40 at a concentration of 10^9^ CFU/kg for seven days, isolated from Tibetan yak yogurt, increased serum superoxide dismutase (SOD) and catalase activities while reducing malondialdehyde (MDA) levels, a marker of oxidative stress (OS), in DSS-induced colitis mice. Additionally, *L. plantarum* JS19, administered at 10^9^ CFU/kg for seven days, demonstrated a reduction in OS in acute colitis mice, with a 1.4-fold increase in glutathione peroxidase activity and a 1.8-fold decrease in MDA levels in liver tissue compared to colitis mice [9]. Notably, treatment with *L. plantarum* JS19 also significantly decreased serum TNF-α levels and myeloperoxidase (MPO) activity in acute colitis mice. Furthermore, Jang *et al*. [10] reported that administering *L. fermentum* KBL375 at 10^9^ CFU for eight days suppressed protein levels of inflammatory cytokines, including IL-1β, IFN-γ, IL-4, IL-13, IL-6, and TNF-α, as well as chemokines CC motif ligand 2 (CCL2) and CXC motif ligand 1 (CXCL1) in the colon, compared to DSS-treated mice.

(b) Recovery of Gut Barrier and Mucosal Layers in Epithelial Cells: Treatment with a single probiotic strain such as *L. acidophilus*, *B. bifidum*, or *L. rhamnosus* significantly increased trans-epithelial electrical resistance (TEER), which is closely associated with barrier integrity, ranging from 3.5 to 14.1% in H_2_O_2_-treated Caco-2 cell monolayers [11]. Moreover, pretreatment of mice with 5 × 10^8^ CFU of each probiotic strain for seven days resulted in increased expression of tight junction genes occludin, zo-1, and claudin-5, which were reduced by less than 0.5-fold by TNBS compared to the control group [11].

(c) Rebalancing of Gut Microbiota for Gut Homeostasis and SCFA Production: A previous study demonstrated that administering *L. gasseri* 505, isolated from infant feces, at 10^8^ CFU/kg for 11 weeks reduced Staphylococcus composition while increasing the compositions of *Lactobacillus*, *Akkermansia*, and *Bifidobacterium*, accompanied by increased SCFAs in azoxymethane/dextran sodium sulfate (AOM/DSS)-induced colitis-associated mice [12]. Particularly, higher concentrations of propionate and butyrate in fecal samples were observed in mice fed with *L. gasseri* 505 compared to those in AOM/DSS mice.

Based on these studies, these mechanisms may contribute to the alleviation and protection against IBD by probiotics. The protective efficacy of probiotics against intestinal inflammation might be attributed to direct interactions with immune cells or indirect interactions through SCFA production, which interacts with immune cells. SCFAs are also associated with enhanced barrier function through mucin production and the prevention of circulation of pathogen-like molecules. Treatment of an IBD mouse model with *B. longum* Bif10 and *B. breve* Bif11 demonstrated efficacy in antioxidation, anti-inflammation, and maintenance of gut microbiota homeostasis, supporting these findings [13].

Recently, there has been increasing interest in developing optimal combinations of multiple probiotic strains for the treatment of IBD. This approach is pursued to achieve synergistic and/or diverse health benefits that individual strains alone may not provide. A comparative study evaluating different combinations of beneficial bacteria in DSS-induced colitis mice demonstrated that a mixture of *L. reuteri*, *Bacillus coagulans*, *B. longum*, and *Clostridium butyricum* (at a ratio of 1:1:1:1) alleviated colitis more effectively than single probiotic strains [14]. The combination of bacteria significantly alleviated colon shortening compared to the less effective outcomes observed with *L. reuteri* alone and *C. butyricum* alone. While the bacterial combination did not dramatically alter the expression levels of IL-6 and IL-1β compared to each single strain, it significantly increased the expression of cytokines such as IL-10 and tight junction proteins (claudin-1 and occludin), indicating enhanced beneficial effects from the bacterial combination. Additionally, a multi-species probiotic mixture containing *L. acidophilus* LaVK2, *B. bifidum* BbVK3, *Lactococcus lactis* ssp. cremoris NCDC-86, and *Lc. lactis* ssp. *lactis* biovar *diacetylactis* NCDC-60 demonstrated immunoprotective effects in DSS-induced ulcerative colitis (UC) mice [15]. Treatment with this four-strain combination resulted in lower myeloperoxidase (MPO) activity (0.3-fold) and reduced levels of cytokines TNF-α (0.6-fold), IL-6 (0.6-fold), and IFN-γ (0.6-fold) in colon tissue compared to mice treated with mono-species probiotics. Based on these studies, the combination of probiotics appears to offer synergistic beneficial effects on the host, supporting their potential for enhanced therapeutic efficacy in IBD treatment.

The objective of this study is to investigate the efficacy of individual *Limosilactobacillus fermentum* strains IM57, IR51, and IR62, newly isolated from infant feces, in alleviating DSS-induced colitis in mice. Additionally, the study aims to determine whether combining these *L. fermentum* strains can synergistically reduce intestinal inflammation. To achieve this, the antioxidant and anti-inflammatory activities of the individual and combined strains were evaluated in vitro. Furthermore, an in vivo mouse feeding study followed by microbiome analysis was conducted to elucidate the relationship between intestinal microbiota composition and anti-inflammatory effects. This study aims to provide valuable scientific evidence regarding the synergistic effects of probiotic combinations and their potential applications in the treatment of IBD.

## Materials and Methods

### Bacterial Strains

All *Limosilactobacillus* strains used in this study were isolated from Korean infant fecal samples (Institutional Review Board of Korea University, South Korea (KUIRB-2021-0116-02)). All isolates from infant feces were incubated aerobically in de Man, Rogosa and Sharpe (MRS) broth at 37°C for 18 h and subcultured two times before use. The isolates were preserved in MRS broth with 40% (v/v) glycerol at -80°C.

### Acid/bile Tolerance and Intestinal Adhesion Ability

The acid/bile tolerances of the isolates and the adhesion ability of the strains were examined as previously described by Oh *et al*. [16]. In brief, 1% (v/v) of each *Lactobacillus* strain was incubated in 50 mM phosphate-buffered saline (PBS) adjusted to pH 3.0 with 1 N HCl to assess acid tolerance. For bile salt resistance, each strain was incubated in 50 mM PBS (pH 7.0) supplemented with 1% (w/v) oxgall. The survived cells were quantified with standard viable cell counts using pour-plating method on MRS agar.

### Antioxidation Activity

The bacterial culture was centrifuged at 10,000 ×*g*, 4°C for 10 min and the supernatant was collected for antioxidation activity assay. The supernatant sample was filtered through 0.22-μm pore size syringe filter (Thermo Fisher Scientific, USA) and used for assays of the radical scavenging activities and reducing power *via* 2,2’-azino-bis(3-ethylbenzothiazoline-6-sulfonic acid) (ABTS) assay, 2,2-diphenyl-1-picrylhydrazyl (DPPH) assay, the hydroxyl radical scavenging activity assay, and the ferric-reducing antioxidant power (FRAP) assay. The ABTS and DPPH assay were performed as previously described by Oh *et al*. [16], and the hydroxyl radical scavenging activity by the method of Li *et al*. [17]. In addition, the ferric reducing antioxidant power (FRAP) assay was conducted as described by Yu Xiao [18].

### Nitric Oxide Assay for Anti-Inflammatory Activity

RAW 264.7 murine macrophage cells were cultured in Dulbecco’s modified Eagle’s medium (DMEM; Gibco, USA) containing 10% of fetal bovine serum (FBS; Gibco) and 1% of penicillin-streptomycin (PS; Gibco) at 37°C in a humidified 5% CO_2_, 95% air atmosphere. The cells were subcultured every other day. The strains were washed twice with 1×PBS at 10,000 ×*g*, 4°C for 10 min and resuspended in fresh DMEM medium without PS. The suspensions were serially diluted and used three concentrations (10^3^ to 10^5^ CFU/ml). To determine the anti-inflammatory activity, the concentration of NO produced by RAW 264.7 cells treated with selected strains was determined by Griess reaction [19]. 100 μl of RAW 264.7 cells were seeded into 96-well cell culture plate at a density of 1.5×10^4^ cells/well and incubated for 24 h. Afterwards, the sample groups were pretreated with 11 μl of bacterial suspensions (10^3^ to 10^5^ CFU/ml) for 12 h, and stimulated with 12 μl of lipopolysaccharide (LPS; Sigma-aldrich, USA) for another 12 h. The control group was treated only with fresh DMEM medium. After incubation, 70 μl of the supernatants were transferred into a new 96-well plate. And then, 70 μL of Griess reagent was added to each well and reacted for 10 min at room temperature. The optical density was measured at 540 nm using microplate reader (BioTek epoch 2; BioTek Instruments, USA). The production of NO was calculated by comparison with a standard curve of sodium nitrite (0, 1, 2, 4, 8, 16, 32, and 64 μM).

### Whole Genome Sequencing and Comparative Genome Analysis

Genomic DNA extraction of the *L. fermentum* strains were performed as the method of Oh *et al*. [16]. The genomes of the strains were completely sequenced using NovaSeq6000 (Illumina, USA) by Cellkey Inc. (Republic of Korea). The qualified raw sequencing data was filtered using Trimmomatic 0.39 [20], and assembled using Unicycler v0.5.0 [21]. Then, prokka v1.14.6 [22] was used in order to annotate genes and identify coding sequences (CDSs). Circular genome map of the strains was achieved using GenoVi v0.4.3 [23], a software that performs circular genomic representations using Circos [24]. GenoVi automatically calculates and formats graphics CDSs, and calls for DeepNOG [25] to classify them into clusters of orthologous groups of proteins (COGs). Toxin and virulence factors were detected using the virulence factor database (VFDB) and antibiotic resistance genes with the comprehensive antibiotic resistant database (CARD) [26, 27]. For phylogenic and comparative genomic analysis, 62 complete genomes of *L. fermentum* strains were obtained from the NCBI genome database (https://www.ncbi.nlm.nih.gov/genome/). A total of 65 genomes of *L. fermentum* strains (three sequenced in this study and 62 from NCBI) were subject to comparative genome analysis. Phylogenetic tree was constructed based on the Average nucleotide identity (ANI) values of 65 *L. fermentum* strains.

### Animal Experiment

All the animal experimental procedures used in this study were performed and approved by the Institutional Animal Care and Use Committee of Korea University, South Korea (KUIACUC-2023-0089). C57BL/6J mice (male, six-week-old, weighed 18-22 g; Doo Yeol Biotech, Republic of Korea) was used, and maintained under a 12 h light/dark cycle. Animals were supplied with water and normal chow diet freely and acclimatized to laboratory conditions for one week before the experiment. A total 48 mice were randomly divided into six groups of eight animals each: the normal control group (CON; normal diet), DSS group (DSS; DSS with normal diet), *L. fermentum* IM57 group (IM57; DSS with *L. fermentum* IM57), *L. fermentum* IR51 group (IR51; DSS with *L. fermentum* IR51), *L. fermentum* IR62 group (IR62; DSS with *L. fermentum* IR62) and mixture of the strains group (MIX; DSS with the mixture in a ratio of 1:1:1). The sample groups, IM57, IR51, IR62, and MIX, were given 0.2 ml of each strains (10^9^ CFU/ml dissolved in sterilized 1×PBS) by oral gavage once a day for 21 days. The CON and DSS groups were orally administrated 0.2 ml of 1 × PBS instead. Excepting CON group, the five groups, DSS, IM57, IR51, IR62, and MIX, were induced 3% DSS water (molecular weight of 36–50 kDa, MP Biomedical Solon, USA) for seven days at the end of experiment. The experiment design was showed in Fig. 3A. The food intake, body weight, and disease activity index (DAI) were observed every day during the experiment. DAI was recorded based on the body weight loss, stool consistency, and bleeding in the feces (Table S1).

### Fecal Microbiome Analysis

**DNA extraction and sequencing.** Fecal samples were collected and placed in a sterile micro centrifuge tube. Genomic DNA was extracted from fecal samples using QIAamp Fast DNA stool Mini Kit (Qiagen GmbH, Germany) according to the manufacturer’s protocols. PCR amplification and sequencing were performed according to previous study [28] using the Illumina MiSeq Sequencing system (Illumina).

**Bioinformatics.** For taxonomic assignment, the EzBioCloud database was utilized through USEARCH (8.1.1861_linux32) [29], followed by a more precise pair-wise alignment [30]. Detection of chimeras in reads with less than a 97% best-hit similarity rate was carried out using UCHIME [31] and the non-chimeric 16S rRNA database from EzBioCloud. Subsequently, sequence data executed clustering using UCLUST [29]. For correlation analysis, Spearman’s rank correlation of fecal microbial composition with the markers of protective effect of the strains on DSS-induced intestinal inflammation was visualized as heatmap using R package corrplot 0.92.

### Fecal Short Chain Fatty Acids Analysis

For analysis of short chain fatty acids (SCFAs) in the fecal contents of mice, 20 mg of fecal contents were mixed with 50 μl of 50% (v/v) sulfuric acid (Merck Millipore Ltd., Germany) and shaken for 3 min. 11 μl internal standard solution (heptanoic acid) and 200 μl of ethyl ether were added, shaken for 1 min, and sonicated for 10 min. The solution was centrifuged at 10,000 ×*g*, 4°C for 5 min and the 200 μl of supernatant was collected into a new tube. The extraction process was repeated three times, and finally, 200 μl of collected organic phase were used for analyzing the SCFAs. The external standard was a mixture of acetic acid, propionic acid, butyric acid, iso-butyric acid, valeric acid, iso-valeric acid, and hexanoic acid. All standards were purchased from Tokyo Chemical Industry (Japan). Detection was conducted using an Agilent 7890A gas chromatograph (Agilent Technologies, USA) with flame ionization detection (GC-FID).

### Quantitative Real-Time Polymerase Chain Reaction (qRT-PCR)

The RNA was extracted from the supernatant of Raw 264.7 cells treated with the samples and the colon tissue from animal experiment using TRIZOL reagent (Invitrogen, USA) following the manufacturer’s protocols. The concentration of total RNA was measured using a microplate reader with Take3 Microvolume plate (Bio-Tek, USA). PrimeScript RT Master Mix (Perfect Real Time) (TaKaRa Bio, USA) was used for cDNA synthesis. To analyze mRNA expression levels of each factor, quantitative real-time polymerase chain reaction (qRT-PCR) was conducted using the QuantStudio 3 Real-Time PCR System (Applied Biosystems, USA) with the GoTaq qPCR Master Mix (Promega Co., USA). The experiment method of qRT-PCR follows next steps: 50°C for 2 min; denaturation at 95°C for 10 min; 40 amplification cycles of annealing at 95°C for 15 sec; extension at the primer melting temperature (Tm) for 1 min. The mRNA expression level was calculated using the 2^−ΔΔCT^ method and the GAPDH was measured as a housekeeping gene. The PCR primer sequences were listed in Table S2.

### Statistical Analysis

All data are expressed as the mean ± standard deviation (S.D.) of triplicate measurements. The SPSS v.26.0 software (IBM, USA) was utilized for statistical analysis, and the one-way analysis of variance (ANOVA) and *t*-test followed by Duncan's multiple-range tests were used to identify the significance of differences among the groups. *P* values of < 0.05 were considered statistically significant.

## Results

### Stress Survival, Adhesion, Antioxidant and Anti-Inflammatory Activities of *Limosilactobacillus fermentum* Strains

The *Limosilactobacillus fermentum* strains (*Limosilactobacillus fermentum* IM57, *L. fermentum* IR51, and *L. fermentum* IR62) used in this study were isolated from infant feces and showed the range of 98.55 to 99.79 and 94.42 to 108.62 survival rate in acid and bile tolerance, respectively and the range of 65.06 to 69.80 adhesion ability on HT-29 cells (Table S3).

**Fig. 1:**
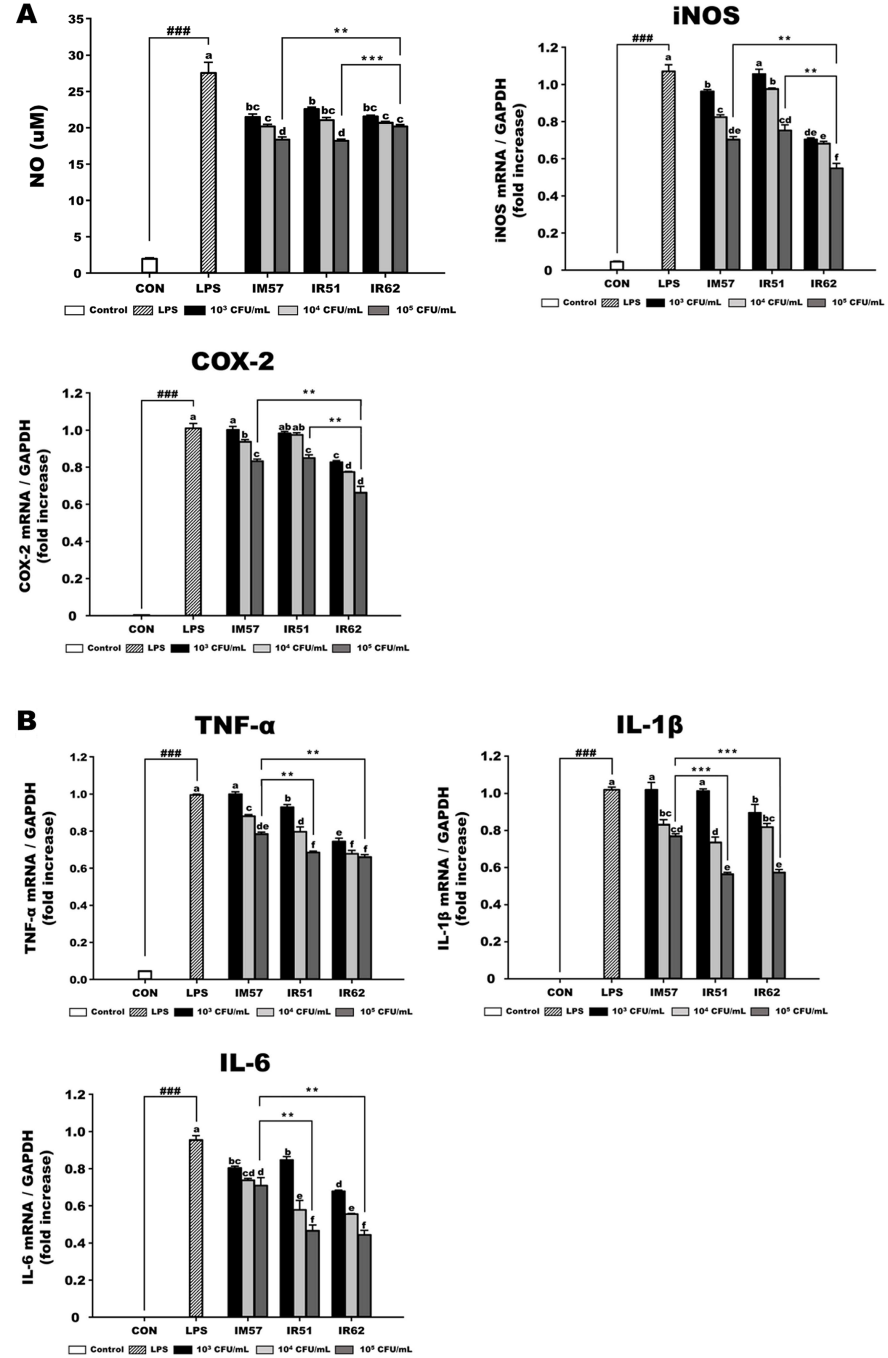
Antioxidant and anti-inflammatory effects of *L. fermentum* strains in LPS-induced RAW 264.7 cells. (**A**) Nitric oxide production and mRNA expression level of iNOS and COX-2 (**B**) mRNA expression level of pro-inflammatory cytokines. CON, cell only; LPS, lipopolysaccharide treated group; IM57, LPS and *L. fermentum* IM57 treated group; IR51, LPS and *L. fermentum* IR51 treated group; IR62, LPS and *L. fermentum* IR62 treated group. Data are expressed as mean ± S.D. (*n* = 3). Bars with different small letters indicate significant differences at *p* < 0.05. The significance of statistics was marked with a hash tag and asterisk (^###^*p* < 0.001 indicate significant differences from the CON, while **p < 0.005, ***p < 0.001 indicate significant differences among each strain).

The antioxidant capacity of the three strains was estimated by measuring the radical scavenging activities and reducing power of their cell-free supernatants. These three strains exhibited ABTS, DPPH and OH radical scavenging activities with the range of the values at 25.96 ± 0.16 to 26.85 ± 0.38, 45.25 ± 0.11 to 52.82 ± 1.71, and 31.09 ± 0.12 to 33.10 ± 0.52, respectively, and the range of their ferric reducing powers were 355.58 ± 0.83 to 357.83± 2.20 (Table 1). Among them, IR51 showed the lowest antioxidant activities. To further understand the antioxidant activities of those three strains, nitric oxide (NO) assay was performed and gene expression levels of inducible nitric oxide synthase (iNOS) and cyclooxygenase-2 (COX-2) were monitored. The inhibitory effects on NO production were shown in LPS-induced RAW 264.7 macrophage cells but their inhibition levels of NO production were similar (Fig. 1A). In addition, the gene expression levels of iNOS and COX-2, which were significantly upregulated by LPS treatment, were also markedly reduced following treatment with the individual strains (Fig. 1A), with the IR62-treated group exhibiting the highest inhibitory effect. Furthermore, an evaluation of the gene expression levels of inflammatory cytokines (TNF-α, IL-1β, and IL-6) demonstrated that treatment with the three strains effectively attenuated LPS-induced increases in cytokine gene expression (Fig. 1B). Consistent with the iNOS and COX-2 results, IR62 showed the most substantial regulatory effect on cytokine gene expression.

### Genomic Characterization of the *L. fermentum* Strains

**Fig. 2:**
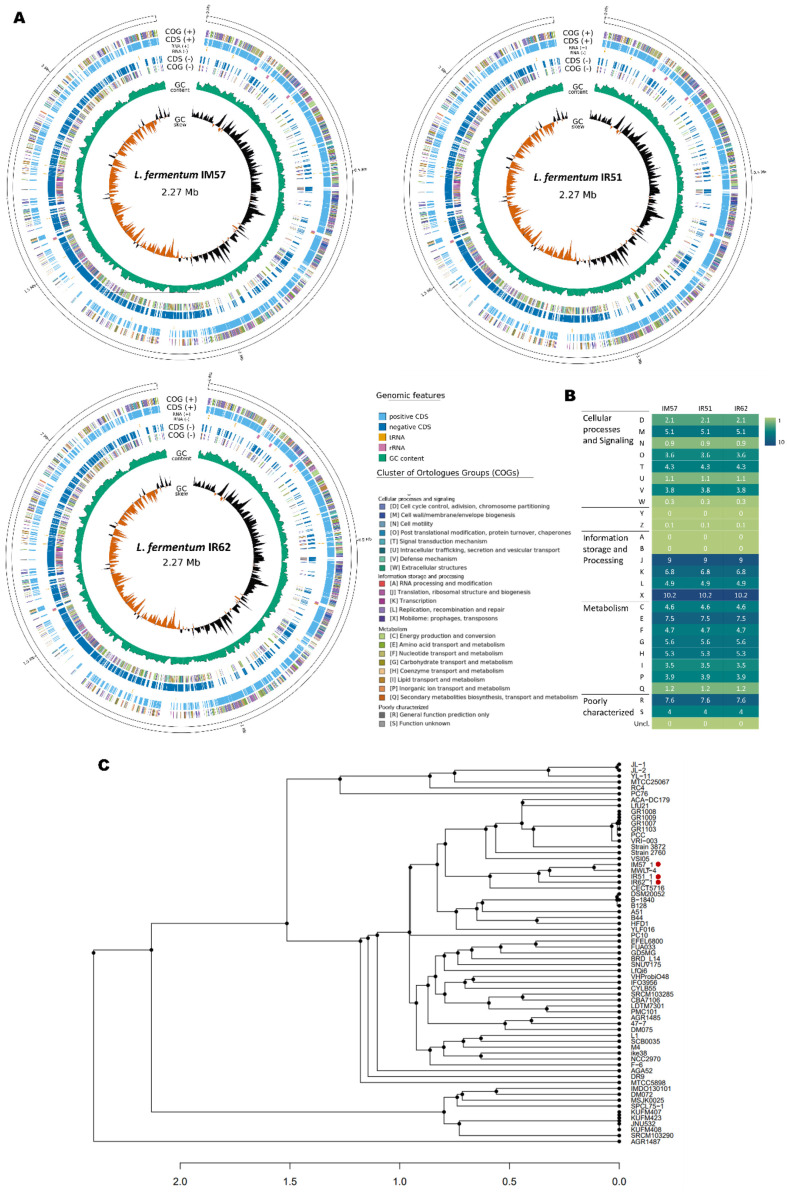
General genomic features of *L. fermentum* strains. (**A**) Circular genome maps of *L. fermentum* strains. From outside to inner of the circle: CDS on forward strand colored by COG category assignment, CDS on reverse strand colored by COG category assignment, GC ratio, and GC skewness. CDS, coding sequence; COG, cluster of orthologous groups; GC, guanine-cytosine. (**B**) COG category classification frequency in percentages. (**C**) The phylogenetic tree based on the ANI values of 65 *L. fermentum* strains.

The genome sequences of the three *L. fermentum* strains exhibited the same features in genome size, GC content, and the number of CDS, rRNA, tRNA (Table S4). The GC content of the strains is 51.36%. The number of ORFs was slightly different among three strains, 3,030 ORFs for IM57 and IR62, and 3,027 ORFs for IR51. However, among the predicted ORFs, functions of 2,193 ORFs (over 67.3%) were predicted with 15 rRNAs and 61 tRNAs. The genomes of *L. fermentum* were predicted by comparison with a protein database, clusters of orthologous genes (COG), and resulted in the same features among three strains as shown in Fig. 2A and 2B. The most abundant COG categories were X, J, and E (mobiliome/translation/amino acid transport and metabolism). 36.3% of genes assigned to functional classifications of COG were assigned to the ‘metabolism’ category and 30.9%to ‘information storage and processing’. Genomic analysis identified genes related to immunomodulation (*groL*, *groS*, *folC*, *dltA*, *dltC*, *dltD*, and *glyG*), antioxidation (*tpx*, *gshAB*, *trxA*, *trxB*, *rib*), and DNA and protein protection and repair (*msrB*), as shown in Table 2. Notably, analysis using VFDB and CARD confirmed the absence of genes associated with toxin production, biofilm formation, virulence factors, or antibiotic resistance. Comparative genomic analysis of total 65 *L. fermentum* strains, including IM57, IR51, and IR62, revealed phylogenetic distinctions among the three strains. IM57 and IR51 were closely clustered within one branch, while IR62 was classified into a separate branch, highlighting genetic divergence (Fig. 2C).

### Impact of the *L. fermentum* Strains in DSS-Induced Colitis Mice

**Fig. 3:**
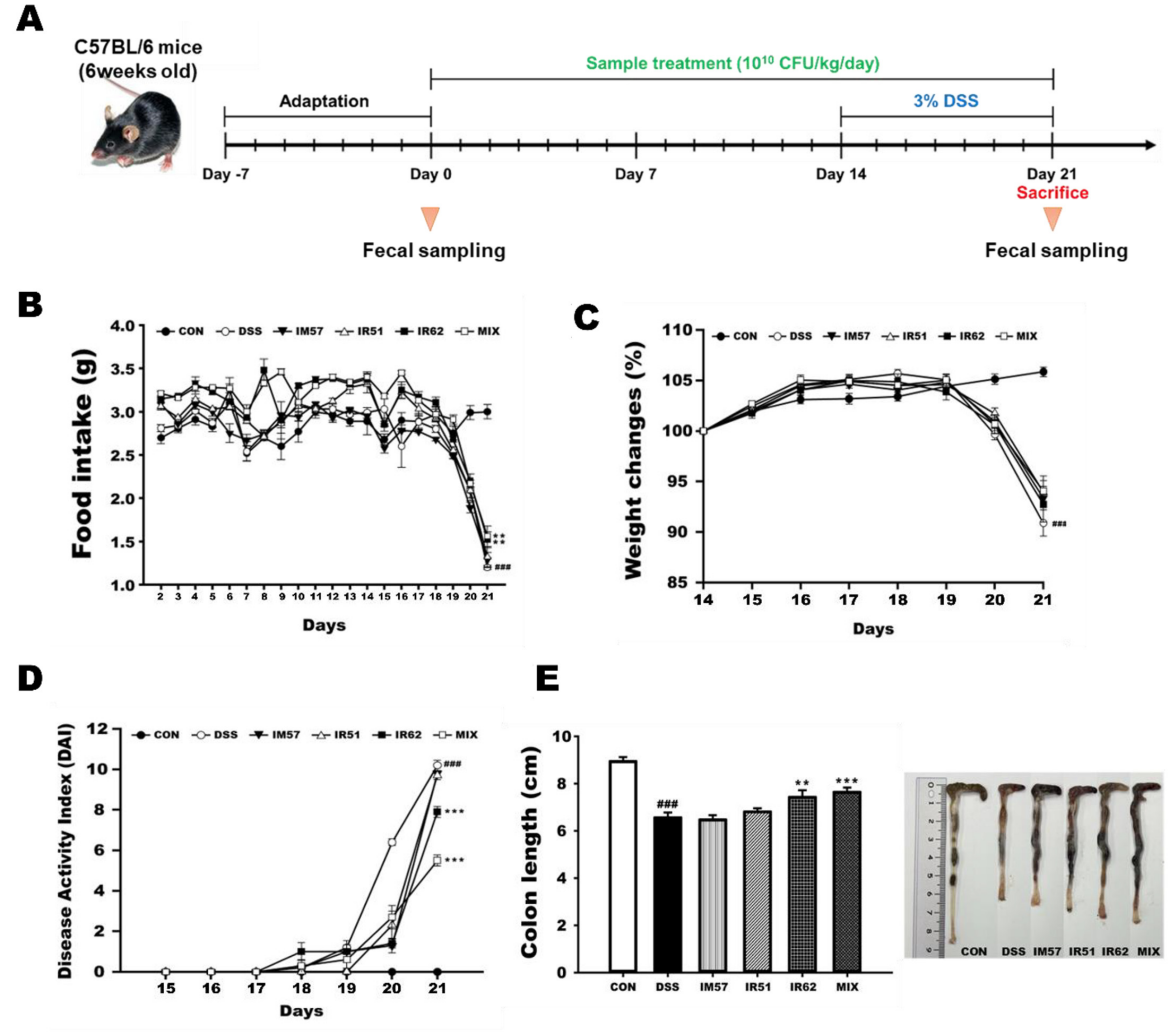
Physiological indexes of DSS-induced colitis mice with treatment of *L. fermentum* strains. (**A**) experiment design, (**B**) food intake, (**C**) weight loss, (**D**) DAI score, and (**E**) colon length. CON, normal group; DSS, colitis model group; IM57, DSS and *L. fermentum* IM57 treated group; IR51, DSS and *L. fermentum* IR51 treated group; IR62, DSS and *L. fermentum* IR62 treated group; MIX, DSS and the mixture of *L. fermentum* strains in a ratio of 1:1:1 treated group. Data are expressed as mean ± S.D. (*n* = 8). The significance of statistics was marked with a hash tag and asterisk (^###^*p* < 0.001 indicate significant differences from the CON, while **p < 0.005, ***p < 0.001 indicate significant differences from the DSS).

The changes of food intake, body weight gain, and disease activity index (DAI) score during the experiment were presented in Fig. 3A-3D. Exposure to DSS led decrease of food intake and body weight gain and increase of DAI score with time-dependent manner, while administration of each of the three *L. fermentum* strains and the mixture of them significantly reduced the DSS-induced changes (*p* < 0.05). Especially the effect against DSS treatment was the greatest in MIX group, followed by IR62-treated group for food intake and DAI score. Moreover, the colon length of DSS group was significantly shorter than that of CON group (*p* < 0.05), but those of IR62 and the mixture-fed groups considerably normalized to that of control (*p* < 0.05), as shown in Fig. 3E.

### Regulation of DSS-Induced Disruption in Gut Microbiota and Metabolites by the *L. fermentum* Strains in Mice

**Fig. 4:**
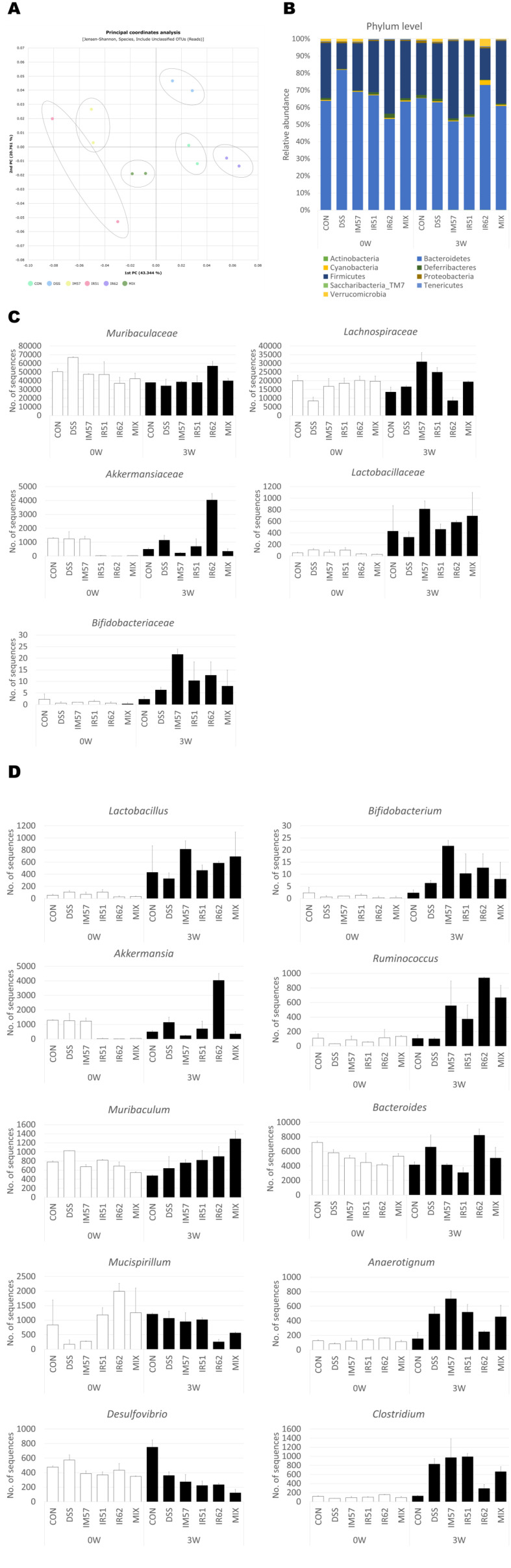
Principal coordinates analysis (PCoA) and microbial compositional changes of representative taxa with treatment of *L. fermentum* strains in DSS-induced colitis mice. (**A**) β-diversity plot using Jensen-Shannon distance and microbial distribution at (**B**) phylum, (**C**) family, and (**D**) genus level.

The gut microbiota composition was confirmed by PCoA and taxonomic analysis. In PCoA analysis, DSS-treated group and the sample groups were in opposite positions relative to the CON group (Fig. 4A). Interestingly, MIX group was located the center of the triangle formed by IM57, IR51, and IR62 groups, particularly IR62 and MIX groups clustered closer to CON group than the other sample-treated groups. As shown in Fig. 4B, there were no differences observed in the relative abundance of two most dominant phyla, *Firmicutes* and *Bacteroidetes*, between before and after treatment in the CON and MIX groups. However, the IM57 and IR51 treatments, along with the DSS treatment, except for IR62, resulted in an increase in the relative abundance of *Firmicutes* and a decrease in those of *Bacteroidetes*. At the family level, *Muribaculaceae* and *Lachnospiraceae* were dominant (Fig. 4C). The abundance of *Muribaculaceae* tended to decrease with the DSS, IM57, and IR51 treatments, while it increased in the IR62 group. Conversely, *Lachnospiraceae* exhibited the opposite trend. Particularly noteworthy is the increase in the abundance of *Akkermansiaceae* in the IR62 group, and the increases observed in Lactobacillaceae and *Bifidobacteria*ceae, as well as in *Lactobacillus* and *Bifidobacteria*, in the IM57 group. Moreover, at genus level, the abundance of *Ruminococcus*, as well as *Akkermansia*, particularly *Akkermansia muciniphila*, dramatically increased, following treatment with IR62 (Fig. 4D). The abundance of *Ruminococcus* was also higher in MIX group than IM57 and IR51 groups. On the contrary, the abundance of *Mucispirillum* and *Anaerotignum* notably decreased in the IR62 and MIX groups, with IR62 exhibiting a particularly pronounced effect in reducing this taxon. Particularly noteworthy was the specific increase in the level of *Muribaculum* in the MIX group. While the abundance of *Muribaculum* decreased in the CON and DSS groups, it increased approximately 2.4-fold after administration of the mixture compared to before administration. In contrast, the increase was 1.31-fold or less in the single strain treated groups.

The concentration of SCFAs as the crucial metabolites of intestinal microorganisms was measured in fecal samples (Table 3). The concentration of total SCFAs such as butyric acid, iso-butyric acid, acetic acid, propionic acid, valeric acid, iso-valeric acid, and caproic acid was significantly decreased in the DSS group compared with the CON group (*p* < 0.05). There was no statistical significance between all of the sample groups and the DSS group for the butyric acid. However, treatment of each strain inhibited the DSS-induced decrease of SCFA concentration. IR62 and MIX groups showed a higher level of all the SCFAs than for DSS group (*p* < 0.05), except for butyric acid. Also, the concentrations of iso-butyric acid, acetic acid, propionic acid, iso-valeric acid, and caproic acid were statistically higher in the IR51 group than for DSS group (*p* < 0.05). Treatment with IM57 had an effect to only the concentration of acetic acid. The highest total concentration of SCFAs was observed as treatment with mixture strains.

### Anti-Inflammatory Effects and Improvement of Intestinal Barrier Function of the *L. fermentum* Strains in DSS-Induced Colitis Mice

**Fig. 5:**
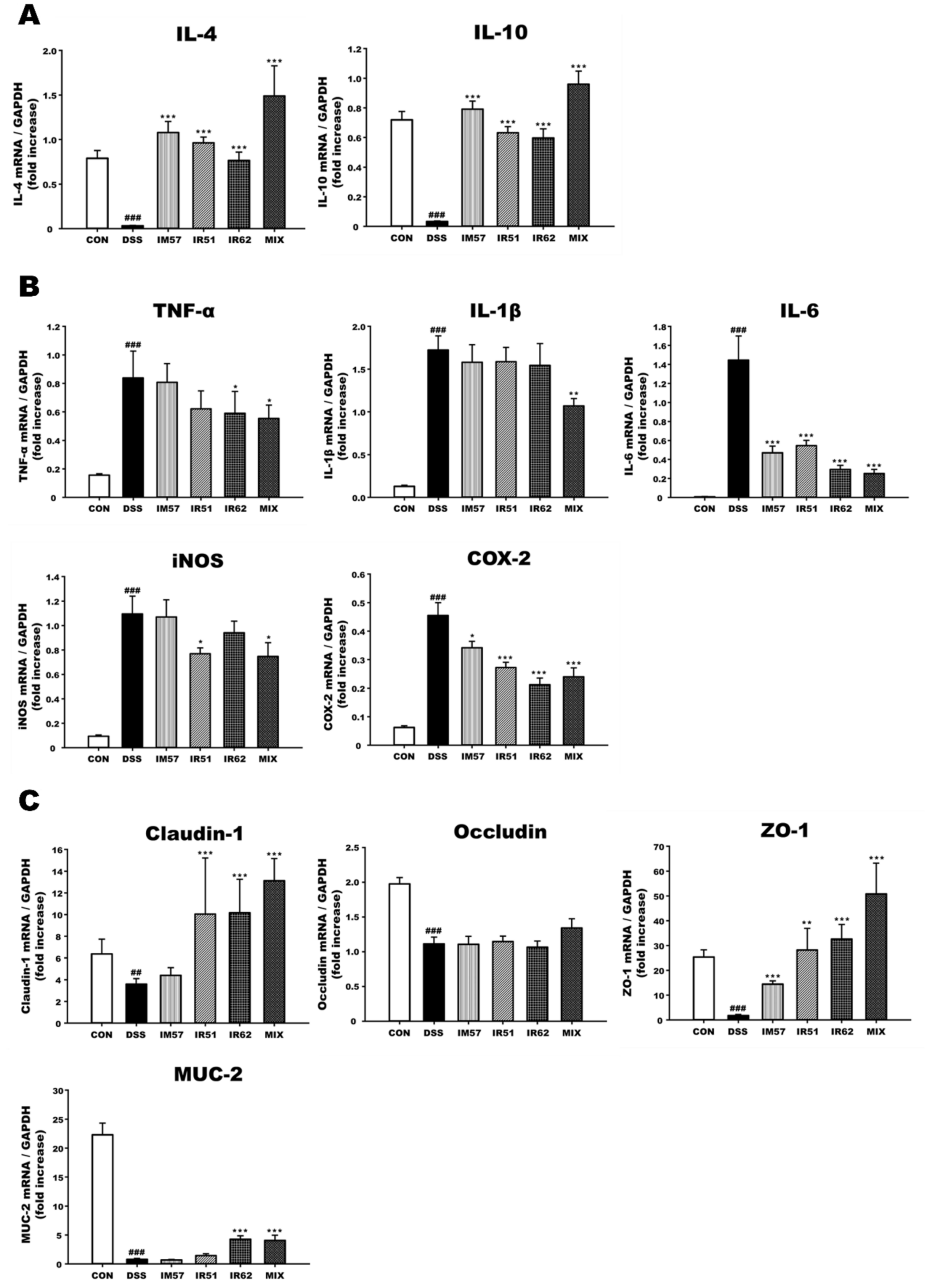
Effects of *L. fermentum* strains on intestinal inflammation and barrier function of DSS-induced colitis mice. (**A**) mRNA expression level of anti-inflammatory cytokines, (**B**) pro-inflammatory cytokines, and (**C**) barrier integrity-related genes in the colon. CON, normal group; DSS, colitis model group; IM57, DSS and *L. fermentum* IM57 treated group; IR51, DSS and *L. fermentum* IR51 treated group; IR62, DSS and *L. fermentum* IR62 treated group; MIX, DSS and the mixture of *L. fermentum* strains in a ratio of 1:1:1 treated group. Data are expressed as mean ± S.D. (*n* = 8). The significance of statistics was marked with a hash tag and asterisk (^###^*p* < 0.001 indicate significant differences from the CON, while *p < 0.05, **p < 0.005, and ***p < 0.001 indicate significant differences from the DSS).

The expression level of anti-inflammatory cytokines (IL-4 and IL-10) and pro-inflammatory cytokines (TNF-α, IL-1β, IL-6, iNOS, and COX-2) in DSS-induced murine colon tissue were presented in Fig. 5A and 5B. In response to DSS, the levels of mRNA expression of IL-4 and IL-10 were significantly decreased and the levels of TNF-α, IL-1β, IL-6 (*p* < 0.05), iNOS, and COX-2 were dramatically increased (*p* < 0.05). The DSS-induced decrease of anti-inflammatory cytokines were significantly inhibited in all sample-treated groups (*p* < 0.05). However, as shown in Fig. 5B, not all the samples regulated the mRNA expression of all pro-inflammatory cytokines. In the TNF-α expression, IR62 and MIX groups showed an inflammation-modulating effect, only MIX group for IL-1β expression, IR51 and MIX groups for iNOS expression, and all samples for IL-6 and COX-2 expression (*p* < 0.05). In particular, treatment with the mixture strains revealed the greatest anti-inflammatory effects in mRNA expressions of all inflammatory mediators (*p* < 0.05).

The mRNA expression levels of tight junction proteins such as occludin, claudin-1, and Zonula occludens-1 (ZO-1) and intestinal mucosa related cytokine Mucin-2 (MUC-2) in DSS-induced murine colon tissue were measured (Fig. 5C). The mRNA expressions of intestinal barrier mediators were significantly dysregulated by DSS treatment (*p* < 0.05). However, the expressions of claudin-1, ZO-1, and MUC-2 were normalized in IR62 and MIX groups (*p* < 0.05). Moreover, treatment with IR51 also upregulated claudin-1 and ZO-1 (*p* < 0.05), and IM57 did only ZO-1 (*p* < 0.05). But, there was no statistical significance of the occludin expression in all the sample groups compared with DSS group.

### Correlation Analysis of Intestinal Microbiota with the Protective Effect of the *L. fermentum* Strains on DSS-Induced Colitis

**Fig. 6:**
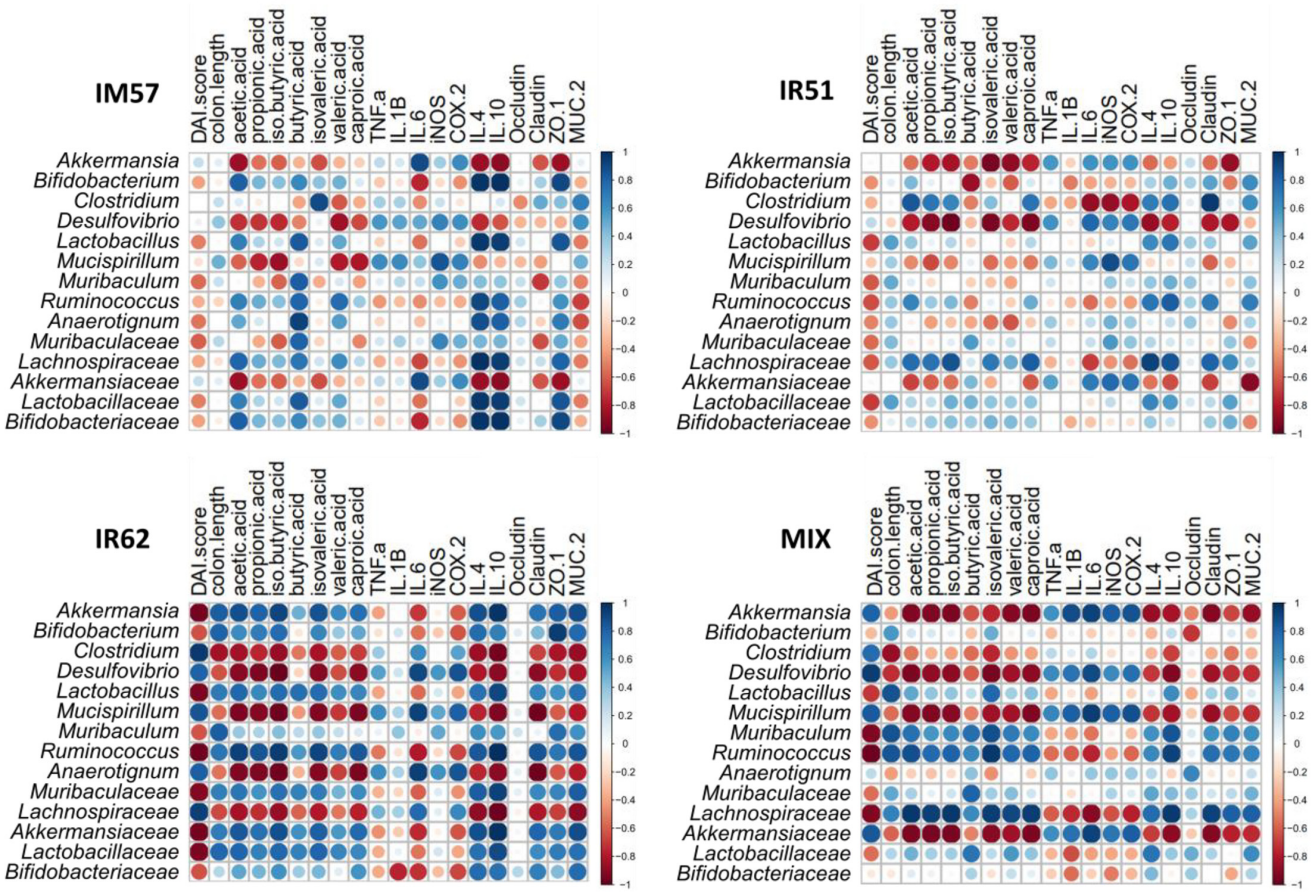
Spearman’s correlation analysis between the representative taxa and intestinal inflammatory markers with treatment of *L. fermentum* strains. CON, normal group; DSS, colitis model group; IM57, DSS and *L. fermentum* IM57 treated group; IR51, DSS and *L. fermentum* IR51 treated group; IR62, DSS and *L. fermentum* IR62 treated group; MIX, DSS and the mixture of *L. fermentum* strains in a ratio of 1:1:1 treated group. The correlation patterns varied across the treatment groups. Notably, the IR62 and MIX groups exhibited stronger correlations between representative taxa and inflammatory markers compared to other groups. In the IR62 group, all selected taxa, except for *Muribaculum*, showed strong correlations with most colitis-associated markers, with the exception of TNF-α, IL-1β, iNOS, and occludin. Interestingly, *Muribaculum* displayed significant correlations with colitis markers exclusively in the MIX group, suggesting a contextdependent role influenced by the combined treatment.

The spearman’s correlation analysis between the microbial composition and the markers of protective effect of the strains on DSS-induced intestinal inflammation was performed (Fig. 6). The correlation trends varied depending on the samples. The correlation between representative taxa and inflammatory markers was observed stronger in the IR62 and MIX groups than others. In IR62 treatment group, the all selected taxa, but not *Muribaculum*, exhibited a high correlation with all colitis-related markers except for TNF-α, IL-1β, iNOS, and occludin. Interestingly, *Muribaculum* was highly correlated with colitis markers only in the MIX group. It is considered that *Muribaculum* may be associated with the prominent effect observed in MIX group regarding the mRNA expression of inflammatory mediators. In MIX group, *Mucispilium* and *Ruminococcus*, which showed strong correlation with colitis-related markers, were likely due to the role of IR62, one of the composed strains in the mixture, while the correlation of *Akkermansia* with colitis-related markers is likely due to IM57 and IR51. These results suggested the microbial composition and inflammatory markers related to DSS-induced colitis due to the administration of IR62 and mixture of three strains to be closely associated.

## Discussion

IBD denotes chronic conditions characterized by inflammation within the gastrointestinal tract caused by various stimuli such as pathogens, damaged cells, or irritants, potentially resulting in various digestive symptoms [32]. IBD occurs in genetically susceptible individuals by an inappropriate immune response to gut microflora. As a therapeutics for IBD treatment, probiotics, which have been widely used for the treatment of various diseases such as aging, inflammation, obesity diabetes, have been explored [33].

Probiotics should be able to survive in and colonize on the gastrointestinal tract maintaining sufficient viability to exert health-promoting effects on the host. In the process of digestion, the viability of probiotic strains undergoes a decline due to gastric juice and hydrochloric acid secreted within the stomach, and bile salts synthesized in the intestine disorganize the structure of the cell membrane [16]. Previously, numerous potential probiotic strains were verified their tolerance against gastric acid and bile salt in vitro. The ability to adhere on intestinal epithelial cells stands as another pivotal criteria for probiotic strains, facilitating their colonization, competitive exclusion of pathogens, and thus provision of host benefits [16]. In this study, the isolated *Limosilactobacillus fermentum* IM57, IR51, and IR62 exhibited great tolerance to acid and bile and adhesion abilities to HT-29 cells. In addition to these basic requirements for probiotics, probiotics have been researched on the health-related properties and mechanisms targeting the various human diseases, such as improving intestinal and oral health, strengthening the immune system, lowering serum cholesterol levels, and anti-diabetic properties. In particular, extensive literature has elucidated the involvement of probiotics in ameliorating IBD *via* in vitro cell tests, preclinical animal models, and clinical trials [34]. Accumulated data suggest that the antioxidant activity, immune enhancement, anti-inflammatory activity, modulating the microbiome, and strengthening intestinal barrier of probiotics have contributed to the alleviation of IBD [34]. Oxidative stress (OS) caused by ROS damages cell, especially lipids, proteins, and DNA in membrane, leading to intestinal mucosal barrier damage and increase of mucosal permeability [35]. Moreover, NF-κB, a redox-sensitive transcription factor, is activated by OS and stimulates release of inflammatory cytokines, and thus intestinal barrier destruction due to the overexpression of inflammatory factors [36]. The antioxidant capacity involving scavenging free radicals, chelating metal ions, modulating antioxidant enzyme expression of probiotics may attribute to improve the status of IBD. In DSS-induced UC mice, *L. fermentum* ZS40, isolated from yak yogurt, increased serum levels of antioxidant enzymes, superoxide dismutase (SOD) and catalase, and reduced myeloperoxidase and malondialdehyde levels by regulating the NF-κB and MAPK pathways [37]. Also, relief of IBD symptoms was observed along with increased expression of antioxidant enzymes such as SOD2 and TrxR-1, and decreased thioredoxin in *L. fermentum* Lf1-fed DSS colitis mice [38]. The antioxidant potential exhibited by diverse species of *Lactobacillus* and *Bifidobacterium*, and their correlation with IBD through the mitigation of inflammation and modulation of immune responses, have been demonstrated in numerous animal models and clinical trials [39]. IBD is literally a dysregulated immune inflammatory state of the gastrointestinal tract, and changes in immune response cells (Macrophage, Treg cells, and T helper cells) including cytokines (TNF-α, IL-1α, IL-6, and IFN-γ) were actually observed in UC and CD patients [40]. However, Jang *et al*. reported that *L. fermentum* isolated from human feces decreased Th1-, Th2-, and Th17-related cytokine levels and increased IL-10 in the colon tissue of DSS-treated mice by regulating immune responses associated with CD4+CD25+Foxp3+Treg cell and altering gut microbiota composition [41]. Additionally, probiotics can improve the damaged intestinal mucosal barrier integrity. *Bifidobacterium breve* UCC2003 was shown to increase and mucus layer generation genes including *Muc2*, *Muc6*, *Muc5c*, and *Muc4* and *Gja1* and *Gjb8* in the mucosa-associated with DSS colitis [42]. And VSL#3 was also shown to increase the expression of tight junction genes, Zo-1, occludin, and claudin-1 in the colon tissue in TNBS-induced colitis mice [43].

As several studies based on molecular genomic analysis [42], identification of antioxidant and immunomodulatory genes within probiotics enhances our understanding of their biological characteristics in alleviating IBD. Previously, *L. johnsonii* N5, which harbors anti-inflammatory-associated genes such as *groEL*, *groES*, and *folC* in its genome, demonstrated a reduction in the expression of pro-inflammatory cytokines including TNF-α in DSS-induced colitis mice [44]. *GroEL* together with *groES* chaperone system plays folding proteins in an ATP-regulated manner, and *groEL* of *Lactobacillus* was demonstrated to inhibit the production of TNF-α induced by LPS [45]. *FolC*, which is involved in folate metabolism and histamine production, within *Lactobacillus reuteri* also inhibited intestinal inflammation by regulating pro-inflammatory cytokines through PKA and ERK signaling [46]. Furthermore, the *dlt* operon, *dltX*, *dltA*, *dltB*, *dltC*, and *dltD*, showed anti-inflammatory potential with regulating inflammatory cytokines [47]. The antioxidant-associated genes, including *tpx* (encoding thiol peroxidase), *trxA* (encoding thioredoxin) and *trxB* (encoding thioredoxin reductase), *npr* (encoding NADH peroxidase), and *nrdH* (encoding glutaredoxin), were identified in the genome of *L. plantarum* LPJBC5 [47]. These genes were observed to contribute to the preservation of intestinal redox balance, removal of ROS, and neutralization of hydrogen peroxide and ROS, thereby attributing to its antioxidant properties. The *L. fermentum* strains IM57, IR51, and IR62 harbor such antioxidant and immunomodulatory genes, as shown in Table 2. However, each strain exhibited different patterns in the expression levels of cytokines and barrier-associated genes in the colon of DSS-induced mice.

To further understand the strain-specific properties in managing IBD, metagenomic analysis of fecal samples from DSS-induced mice administered with each of the strain and a mixture was conducted. At the family level, *Muribaculaceae* and *Lachnospiraceae* were dominant, with the abundance of *Muribaculaceae* decreasing in all treated groups, including those treated with DSS, compared to before treatment. However, it increased only by pretreatment with IR62. The abundance of *Akkermansia*cea also showed the largest increase with treatment of IR62. *Muribaculaceae*, a family within the phylum *Bacteroidetes*, has been associated with the regulation of immune cells, leading to a reduction in the levels of pro-inflammatory cytokines [48]. However, *Lachnospiraceae* was increased in DSS, IM57, and IR51 groups compared to before treatment, but decreased only by treatment with IR62. The *Lachnospiraceae* family are one of the most abundant bacteria in the human gut which belongs to the core of gut microbiota [49]. Although the *Lachnospiraceae* family contributes beneficial effects concerning SCFA production, several studies reported different taxa of *Lachnospiraceae* was correlated with pathologic status [50]. Specific taxa of *Lachnospiraceae* might promote the inflammation by producing inflammatory mediators and metabolizing sialic acids. Furthermore, at the genus level, *Akkermansia* and *Ruminococcus* exhibited a tendency to increase following treatment with each probiotic strain, particularly after IR62 or MIX treatment. *L. fermentum* has been shown to stimulated mucin production as a protective mechanism against infections [51]. In this study, an upregulation of the MUC2 gene was observed in IR62 and MIX groups, suggesting enhanced mucin synthesis. This increase in mucin could promote the growth of mucin-degrading bacteria, such as *Akkermansia muciniphila*, as an increased abundance of *Akkermansia* in IR62 and MIX groups. *Akkermansia*, known for its strong adherence to the mucus layer and specialized intestinal functions [52], produces SCFAs like acetic acid and propionic acid, which interact with host systems through receptors such as Gpr43 [5], contributing to gut health and immune modulation. Gram-negative genus *Bacteroides* exhibits species-specific characteristics. Certain species have the ability to produce SCFA, particularly propionic acid derived from succinic acid, possibly produced from *L. fermentum*, thereby contributing to anti-inflammatory effects. The abundance of SCFA-producing bacteria such as *Ruminococcus* and *Bacteroides* was higher in IR62 and thus MIX groups than in the DSS group, resulting in a relatively high concentration of SCFAs in the IR62 and MIX groups in this study. Interestingly, treatment with the mixture notably increased the abundance of *Muribaculum*, a member of the *Muribaculaceae* family, as well as Coprococcus. The *Muribaculaceae* family, previously referred to as the S24-7 family, is a dominant bacterial group in the mouse gut. Various studies on colitis in mice have shown that *Muribaculaceae* and *Muribaculum* are negatively correlated with inflammatory markers, including pro-inflammatory cytokines, while positively correlated with intestinal barrier function, such as the expression of tight junction proteins and mucin2 [53]. According to Zhou *et al*., *Muribaculum instestinale* is the dominant species in the murine gut microbiota, and treatment with a mixture of *Clostridium* AP sp000509125, *Bacteroides ovatus*, and *Eubacterium limosum* restored its abundance in DSS-induced mice [53]. Furthermore, previous studies have highlighted the role of these genera in enhancing fecal SCFA levels, with a decrease in *Muribaculaceae* associated with DSS-induced colitis in mice [54]. SCFAs are the crucial metabolites of intestinal microorganisms, and have been studied about its preventive effects of colitis [55]. On the other hands, colitis-related dysbiosis is characterized by an increased microbe of *Mucispirillum* and *Clostridium* group [56]. Notably, an increased abundance of *Mucispirillum* was observed in the DSS group compared to the CON group, while administration of IR62 and the mixture reduced the abundance of *Mucispirillum*. *Mucispirillum* associated with inflammatory markers and colitis, specifically *M. schaedleri*, plays a role in inflammation through regulation of OS, secretion systems, and effector proteins, and modifying the mucosal gene expression of host [57]. The highest transcriptomic indication of an anti-inflammatory effect in the MIX group may be attributed to the highest abundance of *Muribaculum*, a potential pivotal biomarker for managing IBD, possibly resulting from a synergistic effect of the three strains within the mixture. Additionally, an inhibition effect on *Mucispilium* induced by the mixture, originating from the *Mucispilium* inhibition effect of IR62 as one of its component strains, could have contributed. These findings on gut microbiota composition were consistent with the results of correlation analysis, indicating that the composition of intestinal microbiota was significantly associated with markers of DSS-induced colitis in mice.

Previous studies on mixtures of probiotics have mostly investigated the effectiveness of the mixture without comparing it to individual component strains. Of the studies that have compared the mixture to single strains, most have not found the mixture to be significantly more effective than the single strain [58]. But, there is still a lack of research on the potential of multi-strain probiotics of the same species, as well as comparative studies on multi-strain probiotics to single component strains. Nevertheless, previous study has demonstrated that probiotic mixture exhibited enhanced efficacy compared to single probiotics, as evidenced by the *L. rhamnosus* GG and *B. lactis* Bb12 combination being more effective in eradicating *H. pylori* than *L. rhamnosus* GG alone [59]. In this study of probiotic mixture on IBD, the administration of the mixture showed a strong correlation between representative microbes and colitis-related markers. Given that IR62 is one of the component strains of the mixture, its preventive effect against DSS-induced colitis may have influenced the regulation of intestinal microbiota and thus the preventive effect against DSS-induced colitis in the MIX group. Furthermore, the combined effect of the three strains within the mixture in modulating microbial composition may have influenced the regulation of mRNA expression of inflammatory cytokines, particularly with regards to the highest abundance of *Muribaculum* observed in the MIX group. To elucidate the molecular mechanisms underlying the strain-specific probiotics and their synergistic capacity in regulating gut microbiota composition and intestinal inflammation, it is still needed to conduct in-depth analysis of the molecular events associated with each *L. fermentum* strain and the mixture in DSS-induced mice using RNA-sequencing or microarray techniques for further study.

**Fig. 7:**
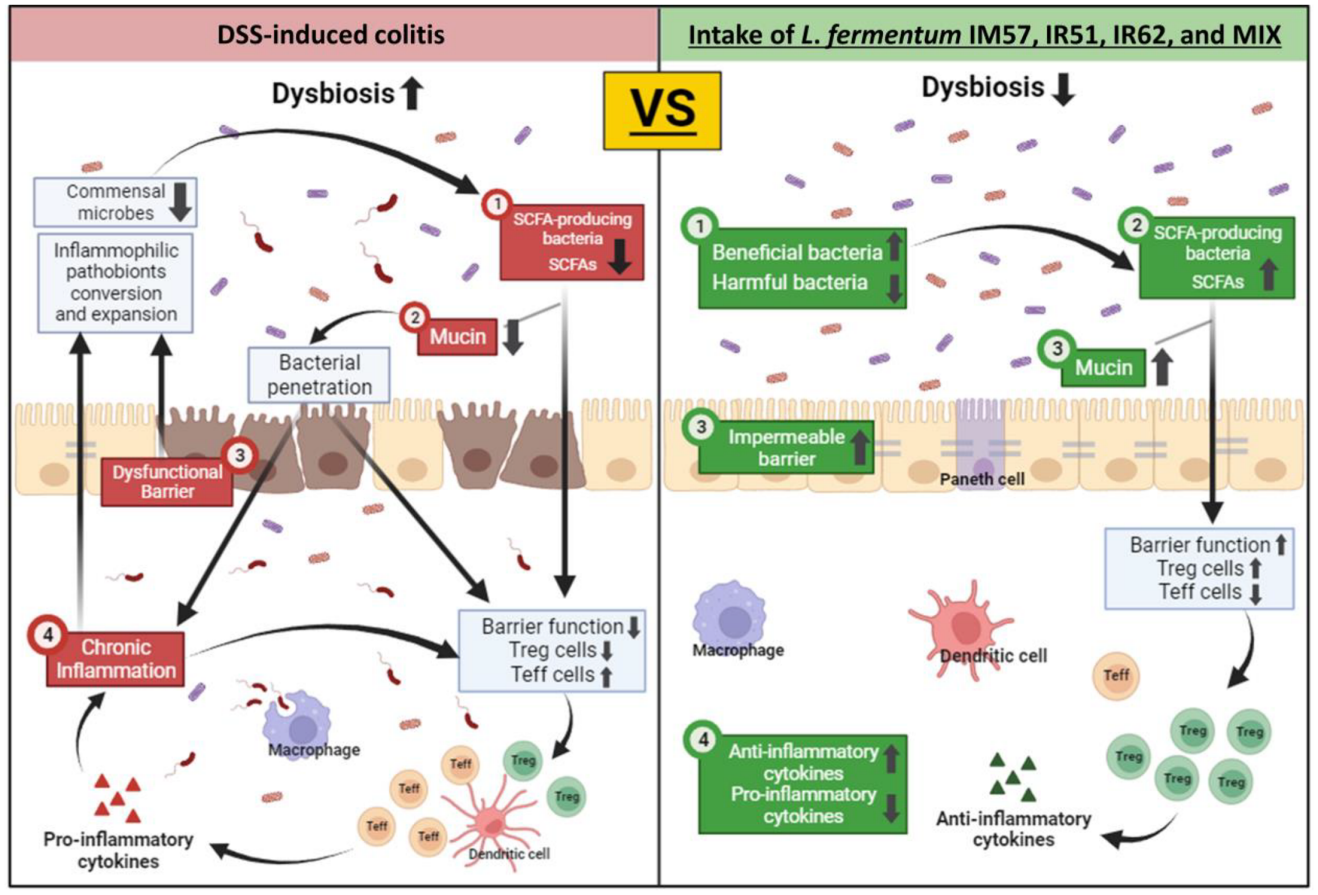
Schematic conclusion of *Limosilactobacillus fermentum* IM57, IR51, IR62 and MIX with protective effect against DSS-induced murine colitis. Three *L. fermentum* strains (IM57, IR51, and IR62), as well as their combined formulation, demonstrated potential protective effects against DSS-induced colitis in murine models. These effects were likely mediated through modulation of the intestinal microbiota, enhanced production of short-chain fatty acids, improved intestinal barrier integrity *via* upregulation of mRNA related to tight junction proteins and mucin production, and the induction of antiinflammatory cytokines, collectively contributing to colitis prevention.

In conclusion, this study indicated that the three *L. fermentum* strains, IM57, IR51, IR62, and the mixture of those strains showed their potential ability to protect the symptoms of DSS-induced murine colitis through modulation of intestinal microbiota (Fig. 7). The microbial composition in the MIX group reflected the combined impact of component single strains. The greatest effect observed in preventing DSS-induced colitis in mice treated with the mixture might be attributed to the balancing of changes in the abundance of bacteria that are typically associated with colitis prevention, while the accumulation of alterations in bacteria positively associated with prevention of DSS-induced colitis. Therefore, the probiotic mixture composed of *L. fermentum* IM57, IR51, and IR62 could play an important role in alleviating IBD.

## Supplemental Materials

Supplementary data for this paper are available on-line only at http://jmb.or.kr.



## Figures and Tables

**Table 1:** Antioxidant activity of *L. fermentum* strains.

	ABTS radical scavenging activity (%)	DPPH radical scavenging activity (%)	OH radical scavenging activity (%)	FRAP value
IM57	26.85 ± 0.38^a^	52.82 ± 1.71^a^	32.15 ± 0.43^ab^	356.42 ± 1.67^a^
IR51	25.96 ± 0.16^b^	45.25 ± 0.11^bc^	31.09 ± 0.12^b^	355.58 ± 0.83^ab^
IR62	26.80 ± 0.19^a^	47.18 ± 4.47^ab^	33.10 ± 0.52^a^	357.83 ± 2.20^a^

ABTS, 2, 2’-Azino-bis (3-ethylbenzothiazoline-6-sulphonic acid) radical scavenging activity; DPPH, 2, 2 Diphenyl-1- picrylhydrazyl radical scavenging activity; FRAP, ferric-reducing antioxidant power; IM57, *L. fermentum* IM57; IM51, *L. fermentum* IR51; IR62, *L. fermentum* IR62.

Data are expressed as mean ± S.D. (*n* = 3). Data with different small letters in same column indicate significant differences at *p* < 0.05.

**Table 2:** Genes related to immunomodulation and antioxidation in genomes of *L. fermentum* strains.

Function	Gene	Annotation	Ref.
Immunomodulation	glyG	Glycosyltransferase GlyG	[60, 61]
	yfkJ	Low molecular weight protein-tyrosine-phosphatase YfkJ	[61]
	csbB	Putative glycosyltransferase CsbB	[60, 61]
	ftsW	putative peptidoglycan glycosyltransferase FtsW	[60, 61]
	rodA	Peptidoglycan glycosyltransferase RodA	[60, 61]
	groL, groS	Chaperon assisting protein folding	[62]
	folC	Folate biosynthesis	[62]
	dltD_1, dltD_2	Protein DltD	[60, 63]
	dltC_1, dltC_2, dltC_3	Membrane protein involved in D-alanine export	[60]
	dltA_1, dltA_2	D-alanine--D-alanyl carrier protein ligase	[60]
	ykoT_1, ykoT_2	putative glycosyltransferase YkoT	[60, 61]
Antioxidation	copB	Copper-exporting P-type ATPase B	[64]
	tpx	Thiol peroxidase	[65]
	gshAB_1, gshAB_2, gshAB_3	Glutathione biosynthesis bifunctional protein GshAB	[64]
	pyrDB	Dihydroorotate dehydrogenase B (NAD(+)), catalytic subunit	[66]
	trxB	Thioredoxin reductase	[64, 66]
	trxA_1, trxA_2, trxA_3	Thioredoxin	[64, 66]
	nrdH	Glutaredoxin-like protein NrdH	[67]
	npr	NADH peroxidase	[66]
	luxS	S-ribosylhomocysteine lyase	[68]
	fimA	Manganese ABC transporter substrate-binding lipoprotein	[66]
	ribBA_1, ribBA_2	Riboflavin biosynthesis protein RibBA	[69]
	ahpC	Alkyl hydroperoxide reductase C	[70]
	ribD	Riboflavin biosynthesis protein RibD	[69]
	pdxK_2	Pyridoxine/pyridoxal/pyridoxamine kinase	[71]
	ribE	Riboflavin synthase	[69]
DNA and protein protection and repair	msrB	Peptide methionine sulfoxide reductase MsrB	[60, 63]

**Table 3:** The concentration of short chain fatty acids in fecal from DSS-induced colitis mice.

	CON	DSS	IM57	IR51	IR62	MIX
Butyric acid	20.97 ± 9.00	11.46 ± 7.41^#^	15.68 ± 5.14	10.94 ± 9.27	15.19 ± 9.40	18.58 ± 7.51
Iso-butyric acid	2.59 ± 0.41	1.87 ± 0.21^###^	1.94 ± 0.17	2.50 ± 0.24***	2.57 ± 0.27***	2.48 ± 0.50*
Acetic acid	201.06 ± 34.55	119.55 ± 18.81^###^	150.70 ± 16.52**	156.52 ± 11.93***	198.12 ± 20.50***	199.72 ± 9.23***
Propionic acid	30.36 ± 5.70	17.29 ± 4.27^###^	20.34 ± 3.96	24.40 ± 3.36**	28.82 ± 5.07***	28.15 ± 3.22***
Valeric acid	4.58 ± 0.84	2.44 ± 0.61^###^	2.70 ± 0.89	2.82 ± 0.81	3.27 ± 0.85*	3.76 ± 0.72**
Iso-valeric acid	3.43 ± 0.49	2.79 ± 0.19^##^	2.99 ± 0.41	3.10 ± 0.27*	3.45 ± 0.27***	5.52 ± 1.87**
Caproic acid	19.23 ± 2.02	8.73 ± 3.24^###^	8.97 ± 2.62	17.51 ± 2.34***	16.74 ± 2.08***	18.88 ± 2.14***
Total	282.21 ± 44.96	164.12 ± 26.98^###^	203.32 ± 21.64**	217.80 ± 20.60***	268.15 ± 29.01***	277.09 ± 16.60***

CON, normal group; DSS, colitis model group; IM57, DSS and *L. fermentum* IM57 treated group; IR51, DSS and *L. fermentum* IR51 treated group; IR62, DSS and *L. fermentum* IR62 treated group; MIX, DSS and the mixture of *L. fermentum* strains in a ratio of 1:1:1 treated group.

Data are expressed as mean ± S.D. (*n* = 8). The significance of statistics was marked with a hash tag and asterisk (^##^*p* < 0.01, ^###^*p* < 0.001 indicate significant differences from the CON, while **p < 0.005, *** *p* < 0.001 indicate significant differences from the DSS).
