# Supplementary material for: Protective Potential of Limosilactobacillus fermentum Strains and Their Mixture on Inflammatory Bowel Disease via Regulating Gut Microbiota in Mice
Source: J Microbiol Biotechnol. 2024 Dec 10;35:e2410009. doi: 10.4014/jmb.2410.10009 (PMC11813365; doi:10.4014/jmb.2410.10009)
Supplement: Supplementary file 1 [file jmb-35-e2410009-supple.pdf]

J. Microbiol. Biotechnol. 2025. 35: e2410009
https://doi.org/10.4014/jmb.2410.10009

Supplementary Tables

Table S1. Scoring criteria for disease activity index.

Score	Body weight loss	Stool consistency	Blood stool
0	none	normal stools	negative fecal occult blood
1	1 - 5%	-	-
2	5 - 10%	loose stools	positive fecal occult blood
3	10 - 20%	-	-
4	> 20%	diarrhea	visible rectal bleeding

Table S2. The list of primer sequences used in this study.

Gene		Primer sequence (5’ - 3’)
TNF-α	Forward	CTGAACTTCGGGGTGATCGG
	Reverse	GGCTTGTCACTCGAATTTTGAGA
IL-1β	Forward	CAACCAACAAGTGATATTCTCCATG
	Reverse	GATCCACACTCTCCAGCTGCA
IL-6	Forward	AAGTCGGAGGCTTAATTACACATGT
	Reverse	CCATTGCACAACTCTTTTCTCATTC
iNOS	Forward	CCCTTCCGAAGTTTCTGGCAGCAGC
	Reverse	GGCTGTCAGAGAGCCTCGTGGCTTTGG
COX-2	Forward	GAAGTCTTTGGTCTGGTGCCT
	Reverse	GCTCCTGCTTGAGTATGTCG
IL-4	Forward	GGTCTCAACCCCCAGCTAGT
	Reverse	GCCGATGATCTCTCTCAAGTGAT
IL-10	Forward	CTTACTGACTGGCATGAGGATCA
	Reverse	GCAGCTCTAGGAGCATGTGG
Occludin	Forward	TCGCTTATCTTGGGAGCCTG
	Reverse	TTCAAAAGGCCTCACGGACA
Claudin	Forward	CCCCATCAATGCCAGGTATG
	Reverse	TTGTTTTCCGGGGACAGGAG
ZO-1	Forward	GTTGGTACGGTGCCCTGAAAGA
	Reverse	GCTGACAGGTAGGACAGACGAT
MUC-2	Forward	ACCTGGAAGGCCCAATCAAG
	Reverse	CAGCGTAGTTGGCACTCTCA
GAPDH	Forward	CATCACTGCCACCCAGAAGACTG
	Reverse	ATGCCAGTGAGCTTCCCGTTCAG

Table S3. Acid and bile acid tolerance and intestinal adhesion activity of *Limosilactobacillus fermentum* strains used in this study.

Strain	Acid tolerance (%)	Bile acid tolerance (%)	Intestinal adhesion activity (%)
IM57	99.79	104.63	65.06
IR51	98.55	108.62	69.26
IR62	98.55	94.42	69.80

IM57, *Limosilactobacillus fermentum* IM57; IM51, *Limosilactobacillus fermentum* IR51; IR62, *Limosilactobacillus fermentum* IR62

Table S4. Genomic information of *Limosilactobacillus fermentum* strains.

Strain	IM57	IR51	IR62
Genomic size (bp)	2,267,305	2,267,305	2,267,305
GC content(%)	51.36	51.36	51.36
Predicted CDS	2193	2193	2193
Number of rRNA genes	15	15	15
Number of tRNA genes	61	61	61

Different letters within a row represent statistically significant differences ($p < 0.05$).

Table S5. α-diversity estimates of microbiota from feces of DSS-induced colitis mice with treatment of *Limosilactobacillus fermentum* strains.

Group	ACE	CHAO	Jackknife	OTUs	NPShannon	Shannon	Simpson	Phylogenetic Diversity	Good's coverage of library (%)
CON	644.253013	656.155747	715.382747	555.3	4.17883433	4.16981	0.031997	787.6666667	99.846073
DSS	636.955478	651.423481	706.141676	559.7	4.27415167	4.26579	0.029581	797.3333333	99.85759333
IM57	634.250633	646.399898	669.892909	573	4.32362567	4.316057	0.02919	807.6666667	99.87522333
IR51	626.953097	641.667633	660.651837	577.3	4.418943	4.412038	0.026774	817.3333333	99.88674367
IR62	654.202122	661.738392	685.614092	605	4.434039	4.427596	0.02625	854	99.90023967
MIX	654.4828	659.533947	684.233183	600.7	4.431775	4.425146	0.027179	850	99.89675233
